# Differential resistance to cell entry by porcine endogenous retrovirus subgroup A in rodent species

**DOI:** 10.1186/1742-4690-4-93

**Published:** 2007-12-14

**Authors:** Giada Mattiuzzo, Magda Matouskova, Yasuhiro Takeuchi

**Affiliations:** 1 Wohl Virion Centre, Division of Infection and Immunity, University College London, W1T 4JF, London, UK

## Abstract

**Background:**

The risk of zoonotic infection by porcine endogenous retroviruses (PERV) has been highlighted in the context of pig-to-human xenotransplantation. The use of receptors for cell entry often determines the host range of retroviruses. A human-tropic PERV subgroup, PERV-A, can enter human cells through either of two homologous multitransmembrane proteins, huPAR-1 and huPAR-2. Here, we characterised human PARs and their homologues in the PERV-A resistant rodent species, mouse and rat (muPAR and ratPAR, respectively).

**Results:**

Upon exogenous expression in PERV-A resistant cells, human and rat PARs, but not muPAR, conferred PERV-A sensitivity. Exogenously expressed ratPAR binds PERV-A Env and allows PERV-A infection with equivalent efficiency to that of huPAR-1. Endogenous ratPAR expression in rat cell lines appeared to be too low for PERV-A infection. In contrast, the presence of Pro at position 109 in muPAR was identified to be the determinant for PERV-A resistance. Pro109. was shown to be located in the second extracellular loop (ECL2) and affected PERV-A Env binding to PAR molecules.

**Conclusion:**

The basis of resistance to PERV-A infection in two rodent species is different. Identification of a single a.a. mutation in muPAR, which is responsible for mouse cell resistance to PERV-A highlighted the importance of ECL-2 for the viral receptor function.

## Background

Pig-to-human xenotransplantation presents potential benefits for treatment of a range of diseases, such as diabetes, neurological disorders and for organ failures, and to alleviate the shortage of human donor organs. Recent advances in genetic engineering of animals, such as the development of pigs devoid of α-galactosyltransferase [1,2], help overcome immunological problems and bring clinical xenotransplantation a step closer to reality. However, zoonotic pathogen transmission is a potential risk and must be controlled (reviewed in [3] and [4]). Although exogenous viruses can be removed from the transplantation source by breeding pigs in specific pathogen-free environments, such techniques cannot eliminate porcine endogenous retroviruses (PERV) present in the pig germ line DNA. Furthermore, pig cells can produce PERV capable of infecting human cells *in vitro *[5-7]. All PERV known to be infectious belong to the gammaretrovirus genus and gammaretroviruses, such as gibbon ape leukaemia virus (GALV) and murine leukaemia virus (MLV), can cause cancer, leukaemia or neurodegeneration. If PERV cross the species barrier, adapt to new human hosts and create epidemics, the risk will be not only to the patient who receives the xenograft, but also to the general public. The recent spread of koala endogenous retrovirus in the koala population represents an example of the hazards associated with gammaretroviral cross-species infection [8].

Three subgroups (A, B and C) of infectious PERV share similar *gag *and *pol *genes, but differ substantially in the *env *gene and therefore in their receptor usage and host range: PERV-A and B, but not C, can infect human cells *in vitro *[9]. All human-tropic PERV isolates derived from primary porcine cells contain at least a part of PERV-A *env *and utilise PERV-A receptors for cell entry. As the greatest threat comes from high-titre, human-tropic recombinant PERV [10-13], such as PERV-A 14/220 isolate [12,13], PERV-A receptors would be the major route for potential PERV transmission to humans. Two PERV-A receptors (PAR) in human cells, called huPAR-1 and huPAR-2, as well as their murine homologue (named muPAR in this study) have been cloned [14]. HuPAR-1 and huPAR-2 are paralogues and their amino acid (a.a.) sequences share 86% homology. The muPAR genomic locus has been previously described as syntenic to the huPAR-2 locus [14], whereas complete sequencing of human and mouse genomes shows that muPAR is syntenic to huPAR-1, not huPAR-2. Our search for PAR homologues in the GenBank genomic sequence database identified homologues syntenic to huPAR-1 and muPAR in all complete genome sequences (chimpanzee, rat, dog, rhesus macaque, cow and horse). A pig cDNA coding for a PAR homologue is functional as a PERV-A receptor [14]. Additional homologues were only found in primate genomes, namely chimpanzee and rhesus macaque, and proved to be syntenic to huPAR-2, while a baboon cDNA closely related to huPAR-2 has been cloned [14]. It is likely that a duplication event gave rise to PAR-2, since the extra copy of PAR appeared after the separation of the primates from other mammalian species. PAR expression has been shown in a wide variety of human tissues by northern blot using a probe detecting both huPAR-1 and huPAR-2 [14]. Our further investigation using EST Profile Viewer [15] has indicated ubiquitous expression of huPAR-1 in different human tissues, whereas huPAR-2 expression appears to be low and limited to certain tissues including placenta, larynx and prostate. Function(s) of PAR other than that as a PERV-A receptor are yet unknown.

The predicted multiple transmembrane structure of PAR proteins and the ubiquitous expression of HuPAR-1 are common characteristics among gammaretrovirus receptors. A number of them have physiological functions as transporters of different substrates [16-20], suggesting that PAR proteins are involved in the transport of unidentified substrates. The host range of retroviruses is often controlled at the cell entry level and fine structural differences in the receptor primary sequences generally determine species-sensitivity to gammaretroviral entry (reviewed in [21]). However, alternative mechanisms to block viral entry have also been described. N-linked glycosylation of the receptor or production of soluble factor(s) can inhibit the receptor function, while suboptimal expression of the functional receptor may not support infection [22-26].

Here we studied the resistance to PERV-A entry in cells of two rodent species, mouse and rat, to better understand the molecular mechanism of PERV-A entry. Implication from our results in host-pathogen interaction is also discussed in the evolutionary context.

## Results

### Resistance of rodent cells against PERV-A infection

Mouse and rat cell lines have been shown to be resistant to PERV-A infection [9,10]. The host range of gammaretroviruses are often determined by the functionality of their receptor genes [21]. Transfection of cDNA for human PAR receptors, huPAR-1 and 2, but not their murine homologue, muPAR, conferred PERV-A infectivity in otherwise resistant rabbit and murine cell lines [14]. Based on these results we hypothesised that the PERV-A resistance of mouse and rat cells may be due to defective mutations for PERV-A receptor function in muPAR and the rat homologue, ratPAR, and that such mutations may be shared in these two rodent species. We set out our initial experiments to test this hypothesis and first cloned a cDNA for rat PAR from PERV-A resistant NRK cells. Its predicted amino acid sequence is almost identical (only 2 a.a. difference in 450 a.a.) to that in the rat genome database [GenBank: XM_343272] and differs from the muPAR sequence by 9.6% (Table 1). MuPAR and ratPAR are similarly distant from huPAR-1 and -2, about 20% mismatch and share 43 rodent-specific mutation (a.a. present in mouse and rat but different from human) in 450 a.a..

**Table 1:** Amino acids identities

			RatPAR
		MuPAR	90.4%
	HuPAR-1	81.1%	79.3%
HuPAR-2	86.1%	79.6%	79.0%

**Figure 1:**
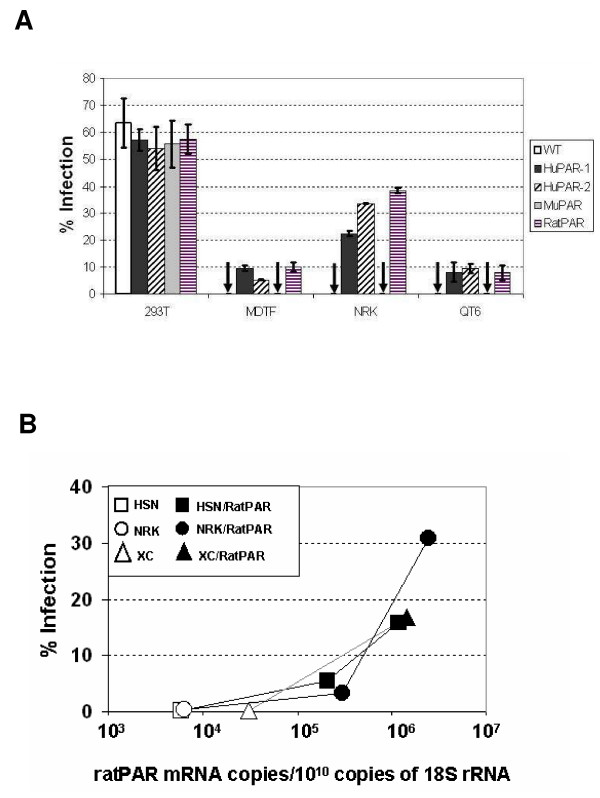
**PERV-A receptor function of HuPARs and their rodent homologues**. **A**. The different cell lines were transduced with the same amount of retroviral vector encoding the HA-tagged receptor genes. Transduced cells were then infected with EGFP(PERV-A). 48 hours post-infection cells were analysed by flow cytometry and the efficiency of infection was determined as percentage of EGFP positive cells. The histograms represent the average ± SEM from three independent experiments. The arrows indicate an infection below detectable levels. **B**. NRK, HSN and XC rat cells were transduced with a retroviral vector encoding the ratPAR gene. Two independent transductions were performed on NRK and HSN cells. The RNA from transduced and untransduced rat cells were extracted. The amount of ratPAR was determined by real time RT-PCR and normalised to equalised copies of 18S rRNA. The results were correlated with the efficiency of EGFP(PERV-A) infection. All the samples were run in duplicate and the experiment repeated at least two times.

Next, we tested the receptor function of rodent PARs in comparison with human PARs. In this assay, all receptors were expressed as C-terminal HA-tagged forms using an MLV-based retroviral vector. This allowed stable PAR expression in various target cells and quantification of their surface expression by immunostaining with an anti-HA antibody. Human 293T, murine MDTF, rat NRK and quail QT6 cells were transduced to express various PARs, so that 50 to 70% of the cells expressed PAR on their surface (see Additional file 1 Fig. S1A). PERV-A infection of cells with or without various PARs were tested using high-titre PERV-A containing an MLV vector genome encoding EGFP [EGFP(PERV-A)] [13] (Fig 1A). The overexpression of any PAR in human 293T cells did not increase the infection efficiency, suggesting that endogenous huPAR expression supports maximal PERV infection in these cells. Despite no PERV-A infection being recorded in MDTF, NRK and QT6 cells without exogenous PAR, these resistant cell lines became susceptible to PERV-A infection upon expression of huPAR molecules (Fig 1A). This result suggests that PERV-A infection is blocked at the entry level and that expression of a functional receptor can overcome this block. MuPAR, unlike huPARs, could not rescue PERV infection when expressed in resistant cell lines (Fig 1A). This result, consistent with the previous report [14], confirmed that muPAR expressed on the cell surface is defective in PERV-A receptor function.

RatPAR, like huPARs and unlike muPAR, allowed PERV-A infection in all the resistant cell lines, including rat NRK cells from which it was derived (Fig 1A). It was suspected that the ratPAR expression level is critical for sensitivity to PERV-A entry. Due to the unavailability of an anti-PAR antibody, it was not possible to investigate endogenous protein expression. Therefore, the amount of ratPAR mRNA was measured by real time RT-PCR in three rat cell lines, NRK, HSN, and XC, before and after exogenous expression of ratPAR. PERV-A infectivity of these cultures is plotted against the ratPAR mRNA level in Fig 1B. Rat cells became PERV-A sensitive when the level of ratPAR mRNA was increased 40–500 fold by exogenously expressing ratPAR. The endogenous expression level of ratPAR therefore appears to be too low to support PERV-A infection, whereas exogenous ratPAR was overexpressed to the level high enough to allow PERV-A entry in rat cells. To demonstrate the dependence of PERV infection on ratPAR expression level, we produced QT6 cell clones with various expression levels of C-terminal HA-tagged ratPAR. PERV-A infection efficiency was dependent on the ratPAR expression level as measured by anti-HA surface staining (see additional file 2 Fig S2). Overall, the mechanism of resistance to PERV-A entry differs between two rodent species, mouse and rat, and the molecular basis of muPAR defect was further investigated.

### Proline 109 in muPAR is responsible for PERV-A resistance

**Figure 2:**
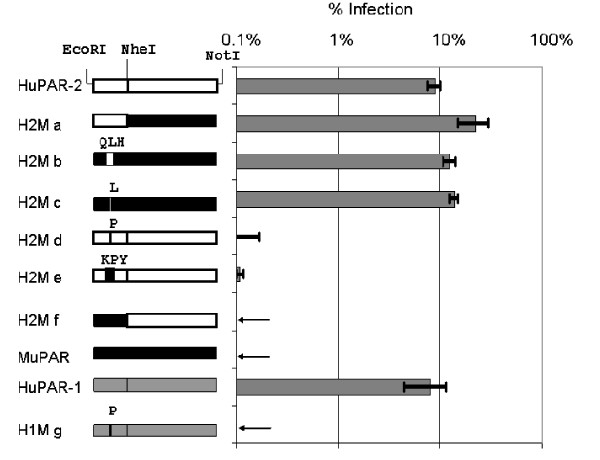
**Identification of critical amino acid residues for PERV-A infection**. HA-tagged chimeric receptors (H2M a-f) between huPAR-2 (white bars) and muPAR (black bars) as well as huPAR-1 (grey bar) and the mutant H1M g were introduced into QT6 cells by MLV-based retroviral vectors. 50–70% of the QT6 cell population showed PAR expression as confirmed by anti-HA staining. These cultures were infected with EGFP(PERV-A). Cells were harvested 48 hours later and PERV-A infection was measured by flow cytometry as percentage of EGFP-positive cells. Arrows indicate infection below detectable levels. Results are expressed as average ± SEM from three independent experiments.

Few a.a. changes in gammaretrovirus receptors inactivate the receptor function of their homologues in different species resistant to viral infection [27-31]. To identify critical a.a. residues for PERV-A infection in PAR, human-mouse chimeric receptors were constructed. Their PERV-A sensitivity was tested in non-permissive quail QT6 cells by transduction of chimeric PAR in retroviral vectors followed by EGFP(PERV-A) infection (Fig 2). Similar results were, however, obtained using murine MDTF cells (data not shown). Figure 2 summarises infection assay results: among the series of chimeric constructs between huPAR-2 and muPAR, H2M a-c, which contained Leu109 derived from the huPAR-2 sequence, were as sensitive to PERV-A infection as the wild-type huPAR-2. Conversely, H2M d-f, possessing the murine Pro109, conferred either zero (H2M f) or near background (H2M d and e) infection. Similarly, no PERV-A infection was detected for huPAR-1 with a Leu-to-Pro change at position 109 (chimera H1M g). These results demonstrated that single a.a. changes at position 109 from Leu-to-Pro in both huPAR-1 and -2 inactivate their PERV-A receptor function and Pro-to-Leu change in muPAR restores PERV-A sensitivity. The adjacent positions 108 and 110 also have different a.a. between huPARs and muPAR. However, a.a. changes at positions 108 and 110 did not affect PERV-A sensitivity of chimeric constructs either in combination (compare H2M b and c; H2M d and e in Fig 2) or separately (data not shown). Pro109 is therefore solely responsible for the inability of muPAR to support PERV-A infection.

### The critical amino acid at position 109 is located in the second extracellular domain of PAR

**Figure 3:**
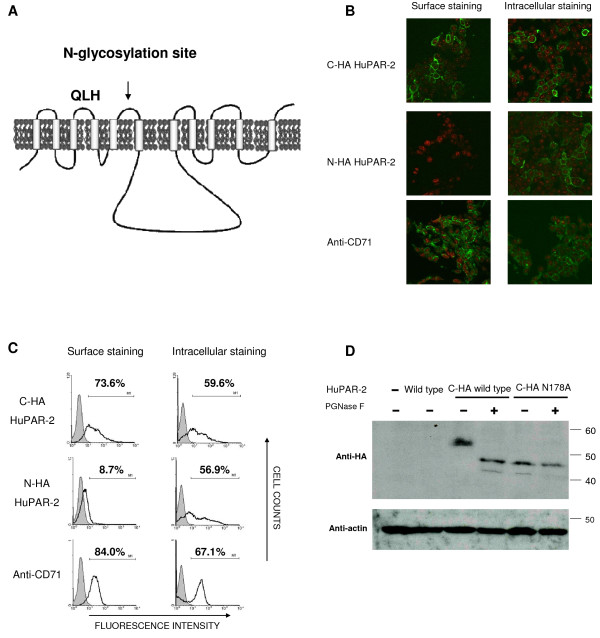
**HuPAR topology**. **A**. HuPAR-2 topology model derived by hydrophobicity algorithms and the experiments described in panel B-D is depicted. **B-C**. HuPAR-2 bearing an N- or C-terminal HA-tag was transiently transfected into 293T cells. After 48 hours cells were treated with saponin (intracellular staining) or without (surface staining). Following immunostaining using an anti-HA antibody and a FITC-conjugated secondary antibody, the samples were visualised either by confocal microscopy (B) or processed by flow cytometry (C). Immunostaining of the cells with anti-human CD71 was used as cell surface protein control. The cells nuclei were counter stained with propidium iodide. **D**. Cell lysates from 293T transiently transfected with an empty pcDNA3 (-), HuPAR-2 (wild type), HA-tagged HuPAR-2 wild type (C-HA wild type) or glycosylation mutant (C-HA N178A) were either treated (+) or untreated (-) with an enzyme removing N-linked oligosaccharide chains (PNGase F) and analysed by western blotting.

A likely mechanism for how a.a. 109 affects the viral receptor function of PAR is that this a.a. is located on the cell surface and controls binding between PAR and PERV-A Env. A previously proposed topology of the PAR molecule has 10 or 11 transmembrane domains (TM) [14]. Our updated transmembrane prediction analysis by TMHMM server v.2.0 [32] suggested a topology with 11 TM, five extracellular loops (ECL), an intracellular N-terminus and extracellular C-terminus (Fig 3A). To validate this topology, huPAR-2 was HA-tagged at its N- or C-terminus. Upon their expression, both the receptors are functional in supporting PERV-A infection in QT6 cells (data not shown). The receptors were transfected into 293T cells and their expression and localisation were studied by immunostaining with or without cell permeabilisation. Localisation of C-terminal HA-tagged huPAR-2 at the cell membrane was visualised by staining both with and without permeabilisation, while N-terminal tagged molecules were visualised only under permeabilised conditions (Fig 3B). Localisation of a fraction of GFP-tagged huPAR-2 to the cell membrane has been previously shown with a major signal also seen intracellularly [14]. In contrast, the less bulky HA-tag used in this study demonstrated predominant membrane localisation of huPAR-2. These stainings were consistently detected by FACS analysis, whereas the staining of N-terminal-tagged molecules without permeabilisation was negative (Fig 3C). Moreover, C-terminal HA tagged huPAR-2 staining was consistent with that obtained with an anti human transferrin receptor (CD71), a protein expressed on the cell surface of active proliferating cells. These results support the transmembrane prediction with 11 TM topology.

Further evidence to support the predicted topology was obtained utilising a glycosylation study. Using NetNGlyc 1.0 software [33], one N-glycosylation site for huPAR-2 at a.a. position 178 is postulated. This prediction agrees with the proposed topology because Asp178 is located in the third ECL (Fig 3A). To test this hypothesis, huPAR-2 harbouring the single a.a. mutation, Asp178 to Ala (N178A), was generated. The construct expressed in QT6 cells supported PERV-A infection (data not shown). Cell lysates of 293T cells transfected with HA-tagged huPAR-2 wild type or the mutant N178A were treated with PNGase F, an enzyme which removes N-linked oligosaccharide chains. The western blot analysis showed a shift of the signal in the wild type huPAR-2 treated with PNGase F from 55 kDa to 48 kDa (Fig 3D). This shift indicated that huPAR-2 carries N-linked oligosaccharide chains. In contrast, the N178A mutant produced 48 kDa bands in both samples with and without PNGaseF treatment (Fig 3D), suggesting that Asp178 is indeed an N-glycosylation site and therefore located in an ECL. Together, these results strongly support the predicted model for the huPAR-2 molecule (Fig 3A). As similar models were also obtained for huPAR-1 and muPAR by transmembrane prediction, various PAR molecules are likely to have the same topology and have a.a.109 in the second ECL.

### Pro109 abrogates binding of PERV-A Env to PAR

**Figure 4:**
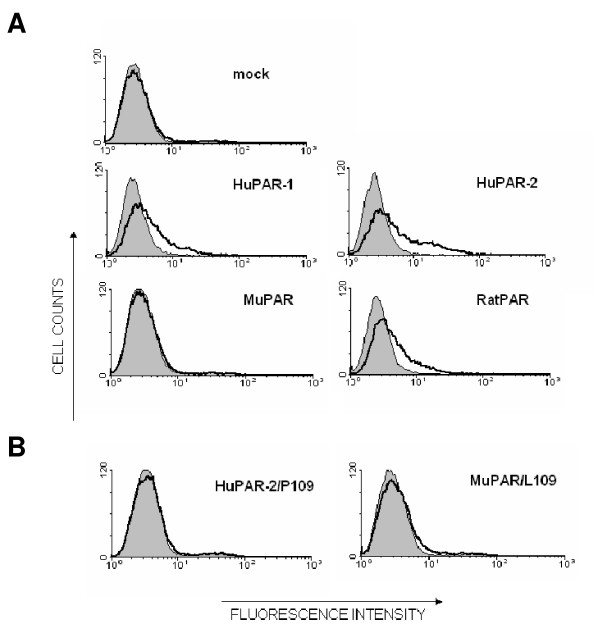
**Envelope binding properties of PAR**. QT6 cells stably expressing HA-tagged receptors huPAR-1, huPAR-2, muPAR and ratPAR (A) or the chimeric receptors huPAR-2 with L109P mutation and muPAR with P109L mutation (B) were incubated with 1 ml of medium (grey filled) or with the supernatant of 293T containing N-terminal c-myc tagged soluble PERV-A 14/220 SU ENV (bold line). The cells were then immunostained with an anti- human c-myc antibody and a PE conjugated anti-mouse IgG secondary antibody. The histograms show a representative result of at least three independent experiments.

To further investigate the mechanism responsible for abrogation of PERV-A infection by Pro109 in muPAR, we analysed the binding properties of the receptors. Parental and receptor-transduced QT6 cells, expressing similar levels of HA-tagged receptors (see Additional file 1 Fig. S1), were incubated with soluble, c-myc-tagged PERV-A envelope protein (mycPERVEnv) and immunostained with an anti-c-myc antibody. No difference was seen between parental QT6 cells incubated in the presence or absence of mycPERVEnv (Fig 4A, mock). However, expression of huPAR-1, huPAR-2 and ratPAR, but not muPAR, produced a shift towards higher fluorescence intensity in the FACS histogram profiles. These results indicate that huPAR-1, huPAR-2 and ratPAR, but not muPAR, can bind soluble PERV-A Env (Fig 4A). To verify whether Pro109 was responsible for the absence of binding of PERV-A Env to muPAR, chimeric receptors huPAR-2 with murine Pro109 (H2M d) and muPAR with human Leu109 (H2M c) were tested in the binding assay. Pro109 completely abrogated the binding of huPAR-2 with soluble PERV-A Env, suggesting that the structure of the second ECL containing Pro109 does not support the interaction between PERV-A Env and the receptor. However the exchange of Pro109 to Leu in muPAR did not rescue the binding of mycPERVEnv (Fig 4B), even if it supported PERV-A infection (Fig 2). This result suggests that other regions in the muPAR molecule, probably involved in the kinetics or affinity of the receptor-Env interaction, are important to achieve a binding efficiency which can be detected in this setting. Alternatively, the discrepancy between binding and function of the mutant receptor H2M c may be caused by a better binding to the trimeric Env form present on viral particles than soluble surface unit monomers.

### Unique structure of PAR ECL2 in murine species

**Figure 5:**
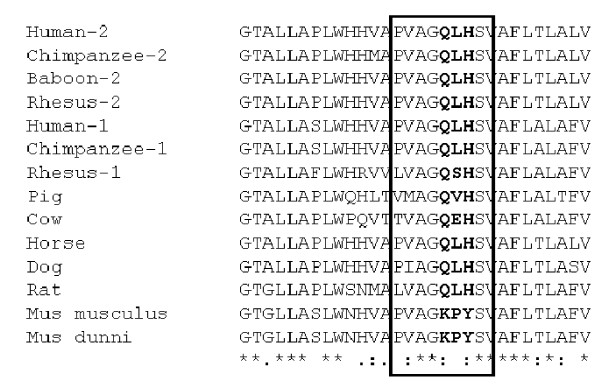
**PAR amino acid sequences alignment**. Amino acid sequences retrieved from Entrez protein database [47] or obtained by direct sequencing of PCR products on genomic DNA were aligned using Clustal W software [48]. *Mus spretus *and *Mus m. castaneus *have the same identical a.a. sequence in the ECL2 (boxed) of other murine species. RatPAR from different rat cell lines have identical a.a. sequences.

PAR a.a. sequences of various species origin were compared. The alignment of ECL2 and the adjacent regions is shown (Fig 5). Two additional murine species, *Mus spretus *and *Mus musculus castaneus*, were also sequenced and in this region, displayed the same a.a. sequence as *Mus musculus *and *Mus dunni*. Pro109 as well as the adjacent Lys108 and Tyr110 are only found in muPAR. In contrast, ratPAR from all 3 cell lines used in this study has the same 3 a.a. triplet, QLH, as huPAR-1 and -2 in the corresponding positions. This confirmed that the receptor function defect is unique in muPAR and ratPAR does not share the same defective mutation as muPAR. Gln108 and His110 are remarkably conserved among non-murine species including rat, a mouse relative within the rodent lineage. Considering a.a. 109 proved to be critical for muPAR interaction with PERV-A, it would be expected to be well conserved among the susceptible species. However, L109 is replaced in two PAR molecules that are functional as PERV-A receptor: Leu-to-Val in porcine PAR [14] and Leu-to-Ser in rhesus PAR-1 (GM and YT, unpublished data). This observation suggests that the a.a. change to Pro109 may cause a substantial conformational change in the ECL2, which results in the inactivation of PERV-A receptor function.

## Discussion

The mechanism of resistance to PERV-A cell entry is different between mouse and rat cells: the murine homologue of PAR (muPAR) is defective in PERV-A receptor function, whereas the rat cell encodes a fully functional PAR protein. RatPAR can rescue PERV-A infection in non-permissive cell lines, including the resistant rat cell lines from which it has been cloned. The PERV-A infection of rat cells upon overexpression of ratPAR is reminiscent of results from a previous study which show that overexpression of amphotropic MLV and GALV receptors from Chinese hamster cells and FeLV-C receptor from MDTF cells, supports viral infection in the cell lines of their origin [26]. This type of resistance to viral infection can be explained by subthreshold levels of receptor expression or stoichiometrically limited masking or interference mechanisms [23-25]. We therefore explored the possibility that a N-glycosylation could mask the receptor and that an inhibitory factor is secreted from rat cells. However, no effect on PERV-A infection by these possible mechanisms was observed (data not shown). The mechanism which determines the threshold level of ratPAR expression for PERV-A infection is currently unclear. However, our results suggest that other component(s) on the cell surface may be responsible for a successful interaction between virus and receptor, as has been previously proposed for other gammaretroviruses [34-36].

The defect in muPAR as a PERV-A receptor is due to the presence of Pro at position 109. Our topology study indicated that a.a.. 109 is most likely to be located in the second extracellular loop (ECL2) and potentially accessible for the direct binding by PERV-A Env. Our binding assay consistently detected soluble PERV-A Env binding to cells expressing 'functional' huPARs and ratPAR, but not muPAR. Furthermore, a Leu-to-Pro mutation at a.a. 109 in huPAR-2 abolished Env binding as well as PERV-A infection, further highlighting the important role of this a.a.. These results identified the ECL2 as the likely target for PERV-A Env binding, leading to PERV-A entry. This, together with recent studies on the determinants in PERV Env for binding and entry [37,38] contribute to better understanding of PERV-receptor interactions. These advances may help develop reagents that block PERV entry, such as neutralizing antibodies [39] and peptides mimicking the receptor.

The amino acid sequence positions 108–110 of muPAR ECL2, KPY instead of QLH, is intriguingly unique in murine species. Since rats share QLH at the corresponding positions with diverse non-murine species including primates, horse and dog, it is likely that murine species acquired 3 mutations after separating from rats. Although we cannot exclude the possibility that these changes are a stochastic evolutionary outcome, it is more likely that certain selective pressure, at least partly, caused these changes. It is tempting to speculate that severe epidemics of PERV-A like viruses which target the ECL2-QLH structure may have selected 'PERV-A-resistant' murine species with KPY. Our result showing that Leu-to-Pro 109 change alone blocks PERV-A infection raises the question why changes are also required at positions 108 and 110. It is possible that all three a.a. changes were required to escape viral attacks in the past. Alternatively the acquisition of Lys108 and Tyr110 by murine species might be required to maintain yet unknown physiological function of PAR while escaping deadly viruses. To further gain insight into this hypothesis, as well as to study involvement of PAR in the possible PERV-A pathology, identification of physiological roles of PAR is warranted.

## Conclusion

Different bases for PERV-A resistance between mice and rats are shown. Expression of endogenous ratPAR in rat cells appear to be under a threshold level to support PERV-A infection. In mice, a single a.a. mutation in muPAR in the ECL2 is responsible for the resistance to PERV-A infection. ECL2 in muPAR has a unique sequence with three a.a. changes compared with a wide range of species. Possible selective pressure may have caused this ECL2 diversion in mice.

## Methods

### Cell lines

Human embryonic kidney 293T cells were maintained in Dulbecco's modified Eagle Medium (DMEM, Gibco) supplemented with 15% fetal bovine serum (FBS, BioSera). Quail QT6 cell [ECACC: 93120831], murine MDTF (*Mus dunni *tail fibroblast), rat NRK [ECACC: 86032002] HSN cells [40] and XC [ECACC: 88120601] were grown in DMEM supplemented with 10% FBS.

### Plasmids and construction of chimeric receptors

The following plasmids have been previously described: murine leukaemia virus (MLV)-based retroviral vectors pCNCG carrying the eGFP gene [41], pCFCR with unique EcoRI site [42], MLV *gagpol *expressor plasmid CMV [43], G protein of vescicular stomatitis virus (VSV-G) expressor plasmid pMDG [44]. Replication competent PERV-A 14/220 plasmid has been previously described [13]. Oligonucleotide primers and probes are listed in Table 2. Soluble surface unit of PERV-A 14/220 Envelope (PERVEnv) was cloned into pCAGGS [45] using the restriction sites BglII and NheI and a c-myc tag has been introduced at N-terminus of PERVEnv using primers G1 and G2 (mycPERVEnv). The sequence of human tissue plasminogen activator leader has been introduced in frame upstream to the c-myc tag by PCR of the construct PEE14 [46] using primers G23 and G24 bearing the enzymatic restriction sites KpnI and BglII, respectively. HuPAR-2 was tagged at the N-terminus with influenza virus HA-tag by PCR of the construct pcDNA3/huPAR-2 [14] using KOD HiFi polymerase (Novagen) and the primers G21 and G22. C-terminal HA-tagged HuPAR-2 was obtained by PCR using the primers G3 and G4 and introduced into pcDNA3 using EcoRI and NotI restriction sites. These primers introduced the Kozak sequence at the ATG of the receptor and the HA-tag in the C-terminus downstream of a HindIII restriction site. Using EcoRI and NotI restriction sites, the HA-tagged receptor was introduced again into pcDNA3. In this way the resulting plasmid pcDNA3/huPAR-2HA contains two HindIII restriction sites, one in pcDNA3 and the other introduced in frame upstream of the HA-tag using the 3' primer. HA-tagged huPAR-1 and muPAR genes were obtained by PCR of constructs pcDNA3/huPAR-1 and pcDNA3/muPAR [14] with the primer pairs G3;G5 (huPAR-1) and G6;G7 (muPAR). Using the HindIII restriction site present in the reverse primers, huPAR-1 and muPAR were cloned into pcDNA3/huPAR-2HA upstream of the HA-tag. All the HA-tagged receptors were also subcloned into the retroviral vector pCFCR using EcoRI and NotI restriction sites.

**Table 2:** Primers and probes used in this study

Name	Sequence (5' → 3')
G1	AGC TGG AGA TCT^a ^GAG CAG AAA CTC ATC TCT GAA GAG GAT CTG^g ^CTT GTG ACC AGT CCG AAC TCC CAT AAA CCC TTA TCT CTC ACC
G2	ATG TTC TTA GCT AGC^b ^CTA TTC ATC AAG GAT TGC TTT TTC CGG
G3	GAT TGA T GA ATT C^d ^AC CAC CAT GG^i^C AGC ACC CAC G
G4	GAT CTT GCG GCC GC^e^T CA A GCG TAT TCT GGA ACA TCG TAT GGG TA^h ^A AGC TT^c^G GGG CCA CAG GGG TCT ACA CAG TCC TTT CTG CTT TG
G5	GAA GGT AAG CTT^c ^GGA GTC ACA GGG GTC
G6	GAT TGA T GA ATT C^d ^AC CAC CAT GG^i^C AGC ACC TCC G
G7	GAA GGT AAG CTT^c ^GAG GCC ACA CTG GTC
G8	CGT GGC ATC TAG ATT AAG CTT^c ^GGG GCC ACA GGG GTC
G9	TTG CAC TAG GGC TAG CAC ACA GG
G10	CCT GTG TGC TAG CCC TAG TGC AA
G11	TAG GAA GGC CAC AGA GTA CGG CTT CCC TGC CAC TGG GGC
G12	GCC CCA GTG GCA GGG AAG CCG TAC TCT GTG GCC TTC CTA
G13	TAG GAA GGC CAC AGA GTG GGG CTG CCC TGC CAC TGG GGC
G14	GCC CCA GTG GCA GGG CAG CCC CAC TCT GTG GCC TTC CTA
G15	TAG GAA GGC CAC TGA GTG GAG CTG TCC TGC CAC TGG GGC
G16	GCC CCA GTG GCA GGA CAG CTC CAC TCA GTG GCC TTC CTA
G17	TAG GAA GGC CAC CGA GTA GAG CTT TCC TGC CAC TGG GGC
G18	GCC CCA GTG GCA GGA AAG CTC TAC TCG GTG GCC TTC CTA
G19	AGA GGT GCC AGC GGT GGG CGC T
G20	AGC GCC CAC CGC TGG CAC CTC T
G21	TTA CAA GAA TTC^d ^GCC ACC ATG G^i^TT TAC CCA TAC GAT GTT CCA GAT TAC GCT^h ^GCA GCA CCC ACG CTG GGC CGT CTG GTG CTG A
G22	GAT CTT AA G CGG CCG C^e^TC AGG GGC CAC AGG GGT CTA
G23	GCC AGA GGA GGT ACC^f ^GCC ACC ATG GAT GCA ATG AAG AGA G
G24	GGG TAA GAT CT^a^G GCT CCT CTT CTG AAT CGG GCA TGG ATT TCC TGG CTG GGC
M1	GAT TGA T GA ATT C^d ^AC CAC CATG G^i^CA GCA CC
M2	TGA CTG A GC GGC CGC^e ^TCA AGG GCC ACA CTG ATC CAC
M3	GCA GGT AAG CTT^c ^AGG GCC ACA CTG ATC
M4	CTC ACT CCT TTA CAC TAC AC
M5	CAA CCC ATT GGA TGA AGA TG
Q1	TCA AGG TGT CTC CCA TCA ATT TC
Q2	CGT CAA CAC CCA AAA GAA TGT G
Q3	TCG AGG CCC TGT AAT TGG AA
Q4	CCC TCC AAT GGA TCC TCG TT
ZF	TAC CTG GTT GAT CCT GCC AGT A
ZR	TTA CGA CTT TTA CTT CCT CTA GAT AG
PR	CTG AGC GTT TCT CTG
P18	AGT CCA CTT TAA ATC CTT

^a ^BglII, ^b ^NheI, ^c ^HindIII; ^d ^EcoRI; ^e ^NotI; ^f ^KpnI, ^g ^Human c-myc tag; ^h ^influenza virus HA tag, ^i ^Kozak sequence.

An NheI restriction site was introduced into huPAR-2 at the site corresponding to that in muPAR [Genbank: AK008081, nucleotide 805] by two-step PCR using primers G3;G9 and G10;G8, then G3;G8, where primers G9 and G10 contain the nucleotide change. Primer G8 includes a HindIII restriction site which allows the cloning of the mutant receptor into pcDNA3/huPAR-2HA. Chimeric receptors H2M a and f were obtained by mix-and-match cloning between huPAR-2 and muPAR using the restriction sites EcoRI and NheI. The other huPAR-2-derived chimeric receptors were produced in a similar way using mutagenesis primers G11;G12 (H2M e) and G13;G14 (H2M d) in association with the primers G3;G8. Similarly, muPAR-derived chimeric receptors were produced using primers G15;G16 (H2M b) and G17;G18 (H2M c) in combination with primers G6;G7.

The N178A mutation in huPAR-2 was introduced by PCR-mutagenesis using the primers G19;G20 in association with the primers G3;G8 and the mutant huPAR-2 was cloned into a partially digested pcDNA3/huPAR2HA using EcoRI and HindIII restriction sites.

The mutant huPAR-1 carrying a proline at position 109 (H1M g) was generated by PCR-mutagenesis using the primers G13;G14 in combination with the primers G3;G5.

All the PCRs described above were performed using KOD HiFi polymerase in accordance with manufacturer's instructions. Chimeric receptors were verified by sequencing based on a modification of the Sanger method and analysed using the CEQ 8000 DNA Sequencer (Beckman Coulter).

### Cloning of rat PERV-A receptor

Total RNA from NRK cells was extracted using the RNeasy kit (Qiagen) and incubated with 5 U of RNase-free DNase (Promega) for 30 min at 37°C. First strand cDNA was produced by incubation of 2 μg of DNase-treated RNA with 200 U of Moloney MLV Reverse Transcriptase (Promega), 1 μg of random primers (Promega), 20 U of RNasin Ribonuclease Inhibitor (Promega), 1 mM dNTPs (Qiagen) in a final volume of 20 μl for 10 min at 25°C, 1 hr at 42°C and an inactivation step of 10 min at 70°C. The ratPAR coding sequence was then amplified using HotStart polymerase (Qiagen) and primers M1;M2 with PCR conditions: 95°C 30 sec, 52°C 30 sec, 72°C 90 sec. Primers M1 and M2 were designed to anneal to the rat homologue of huPAR-1 [Genbank: XM_343272]. The M1 primer introduced the Kozak sequence in front of the ATG of the receptor. The PCR product was cloned into pcDNA3 using EcoRI and NotI restriction sites present in the primers. HA-tagged C-terminal ratPAR was obtained by PCR using KOD HiFi polymerase and the primers M1;M3 which contain the HindIII restriction site, and introduced into pcDNA/huPAR-2HA. This product was then subcloned into pCFCR.

### Transfection, virus production and infection

Transfection of huPAR-2 (N- or C- terminal HA-tagged or N178A mutant) was performed on confluent 293T in a 6-well plate using 4 μl of FuGene-6 reagent (Roche) and 1 μg of plasmid.

Viral particles carrying the receptor genes were produced by co-transfection of 3.5 μg of three plasmids, CMVi for MLV Gag-Pol, MDG for VSV-G and MLV vector genome pCFCR carrying the receptor gene (ratio 1:1:1.5) on confluent 293T cells in 100 mm-dish using 18 μl of FuGene-6 reagent (Roche). Cells were washed 24 hours later and at 48 and 72 hours the supernatant containing viral particles were harvested and passed through a 0.45 μm filter (Millipore). A replication-competent PERV-A 14/220 expressing the reporter gene EGFP, EGFP(PERV-A), was produced as follows. A similar three plasmid transfection on 293T cells was performed using pCNCG instead of pCFCR in order to produce MLV/EGFP particles. The virus-containing supernatant was used to transduce 293T cells. The stable EGFP-expressing 293T cells were then transfected using FuGene-6 with the replication competent PERV-A 14/220 plasmid. The titer of EGFP(PERV-A) viral particles was assessed by infection of 1 × 10^5 ^293T seeded in a 6-well plate using serial dilutions of the supernatant. After two months the titer was stable at 2 × 10^5 ^EGFP 293T transducing units/mL.

The receptor transduction and EGFP(PERV-A) infection were performed as follows: 5 × 10^4 ^target cells were seeded in a 12-well plate and the day after, 500 μl of virus-containing supernatant was added. Receptor or EGFP expression was verified 48 hours post transduction/infection by flow cytometry analysis.

### Flow Cytometry analysis

Cells transfected or transduced with HA-tagged PAR were detached with PBS-5 mM EDTA and blocked by incubation for 30 min in PBS-10% FBS on ice. The cells were washed twice in PBS, resuspended in PBS-2% FBS containing 1:100 dilution of mouse monoclonal antibody HA.11 (Covance) or 1 μg of mouse monoclonal anti-human CD71 antibody (Santa Cruz) and incubated for 1 hour at 4°C. After two washes with PBS-2% FBS, the cells were incubated with 1:200 dilution of the secondary antibody anti- mouse IgG fluorescein isothiocyanate (FITC)-conjugate (Jackson Immunoresearch) in PBS-2% FBS for 45 min at 4°C. Cells were washed three times and resuspended in PBS. To assess EGFP(PERV-A) infection efficiency, 48 hours post-infection cells were harvested and resuspended in PBS. All the samples were processed on a FACScan cytometer (Becton-Dickinson) and analysed using CellQuest software.

### Immunofluorescence microscopy

One day post transfection, 293T expressing HA-tagged huPAR-2 were seeded on cover slides and incubated for further 48 hours. The cells were fixed by incubation with 4% paraformaldehyde (Sigma) in PBS for 20 min at room temperature. The permeabilized samples were obtained by incubation with PBS-0.1% saponin (Fluka) for 10 min at room temperature. For the permeabilized samples, 0.1% saponin was added during all antibody incubations. All slides were washed in PBS and placed on a 30 μl drop of PBS-1% FBS containing antibody HA.11 (dilution 1:100) or anti-human CD71 antibody (dilution 1:50) for 1 hr at 37°C in a humidified chamber. Cells were then washed three times with PBS and the slides placed in a 30 μl drop of PBS-1% FBS containing the secondary antibody FITC-conjugated anti-mouse IgG (dilution 1:100) for 45 min at 37°C in a humidified chamber. After three washes, the cover slides were mounted in Vecta Shield mounting medium containing propidium iodide (Vector Laboratories). Images were collected using a DM IRE2 confocal microscope (Leica).

### Glycosylation assay

293T cells transfected with wild type huPAR-2 or N178A mutant were harvested, washed and incubated in RIPA lysis buffer (50 mM TRIS-HCl pH 7.5, 150 mM NaCl, 1% Igepal CA-630, 0.5% deoxycholic acid, 0.1% SDS, 1% Triton ×-100) in the presence of protease inhibitors (Complete mini, Roche) for 30 min on ice. The cell lysates were then digested with 1500 U of N-glycosidase F enzyme (PGNase F, New England Biolabs) at 37°C for 2 hrs. Proteins from digested and undigested samples were separated by SDS polyacrylamide (BioRad) electrophoresis (SDS-PAGE) and transferred to PVDF membrane (Amersham Biosciences) by using a semi-dry blotting system (Amersham Biosciences). The membrane was blocked in PBS-5% non-fat skimmed milk powder (Oxoid) and then probed for 1 hr at room temperature with the HA.11 monoclonal antibody diluted 1:1000 in PBS-2% milk, followed by incubation with an anti-mouse IgG conjugated with horseradish peroxidase (Dako, dilution 1:10,000 in PBS-2% milk) for 30 min at room temperature. Signals were detected by incubation with ECL chemiluminescence reagent (Amersham Biosciences) and exposure to x-ray film (Hyperfilm, Amersham Biosciences). To control for protein loading, the same blots were incubated with mouse anti-human β-actin (Sigma, 1:1000 in PBS-2% milk).

### Soluble Envelope Binding Assay

C-myc tagged PERV-A 14/220 Env was produced by transient transfection of 293T in a 100 mm dish using 18 μl of Fugene-6 (Roche) and 3 μg of myc14/220ENV plasmid. One day post-transfection, medium was replaced with DMEM supplemented with 10% FBS. The supernatant was then harvested at 48 and 72 hours and passed through a 0.45 μm filter. Target cells for binding assay were detached using PBS-5 mM EDTA, washed twice and 1 × 10^6 ^cells for each sample were resuspended in 1 mL of 293T supernatant containing soluble 14/220ENV. After 1 hr incubation at 37°C, the cells were washed twice with PBS-2%FBS and incubated with 100 μl of anti c-myc antibody 9E10 (Santa Cruz Biotechnology, Inc) diluted 1:100 in PBS-2%FBS for 1 hr on ice. The cells were washed twice and incubated for 30 min on ice with a 1:200 dilution of phycoerythrin (PE)-conjugated secondary antibody anti-mouse IgG (Jackson Immunoresearch) in PBS-2%FBS. After two washes with PBS-2%FBS, the cells were resuspended in PBS and analysed by flow cytometry (FACScan, Becton Dickinson).

### Quantitative RT-PCR

Total RNA from cells was extracted using an RNeasy kit (Qiagen) and cleaned using 5 U of RNase-free DNase (Promega) according to the manufacturer's instructions. The RNA was quantified and 2 μg of total RNA was subjected to reverse transcription (RT) as described for the ratPAR cloning. 2.5 μl of the RT reaction were used in the Real-Time PCR using Quantitect Probe PCR Mix (Qiagen) 0.4 μM of each primers (Q1;Q2), 0.2 μM of Fam-Tamra labelled probes (PR) (Sigma). The amount of RNA between each samples was normalized using the housekeeping gene 18S rRNA, primers Q3;Q4 and probe P18. The assay was performed in duplicate using the ABI PRISM 7000. Thermocycling conditions were: 50°C, 2 min; 95°C, 15 min; 40 cycles of 95°C, 15 sec and 60°C, 1 min. The number of copies of each products were calculated from standard curves obtained by serial dilution of the plasmid pCFCR/ratPAR. Part of the 18S mRNA gene were amplified using primers ZF;ZR from human total RNA and cloned into TOPO BLUNT 2 (Invitrogen) following the manufacturer's instruction.

### Genomic PAR sequence analysis

Genomic DNA was extracted from murine MDTF and rat XC, HSN cell cultures using DNeasy Tissue kit (Qiagen). Genomic DNA from *Mus m. castaneus *and *Mus spretus *is a kind gift from Dr. Jiri Hejnar (Academy of Sciences of the Czech Republic, Prague, Czech Republic). Genomic sequences of rodent PAR were amplified by PCR using high fidelity DNA polymerase KOD HiFi according to the manufacturer's instructions and the primers M4;M5 (muPAR) and M1;M2 (ratPAR). The PCR products were directly sequenced.

### Amino acid sequence accession number

The amino acid sequences used in this study are: huPAR-1 [RefSeq: NP_078807] and huPAR-2 [RefSeq: NP_060456], chimpanzee PAR-1 [RefSeq: XP_001156784] and PAR-2 [RefSeq: XP_001164395], Rhesus macaque PAR-1 [RefSeq: XP_001091189] and PAR-2 [RefSeq: XP_001099620], baboon PAR-2 [Swissprot: Q863Y8], dog PAR [RefSeq: XP_532355], horse PAR [RefSeq: XP_001505049], pig PAR [Swissprot: Q863Y7], cow PAR [RefSeq: NP_001069369], muPAR [RefSeq: NP_083919] and ratPAR [RefSeq: NP_001103140].

## Competing interests

The author(s) declare that they have no competing interests.

## Authors' contributions

YT conceived the study. GM and YT designed the experiments and wrote the manuscript. GM carried out the experiments. MM contributed to cloning and initial characterization of ratPAR. All the authors read and approved the final manuscript.

## Supplementary Material

Additional File 1 PERV-A receptors cell surface expression. Expression of C-terminal HA-tagged PAR constructs in QT6 cells was demonstrated by flow cytometry analysis following surface immunostaining with an anti-HA antibody. Click here for file

Additional File 2 RatPAR function as PERV-A receptor depends on its expression on cell surface. PERV-A infection was measured in quail QT6 cell clones expressing different levels of ratPAR. Click here for file

## References

[B1] Phelps CJ, Koike C, Vaught TD, Boone J, Wells KD, Chen SH, Ball S, Specht SM, Polejaeva IA, Monahan JA, Jobst PM, Sharma SB, Lamborn AE, Garst AS, Moore M, Demetris AJ, Rudert WA, Bottino R, Bertera S, Trucco M, Starzl TE, Dai Y, Ayares DL (2003). Production of alpha 1,3-galactosyltransferase-deficient pigs. Science.

[B2] Kolber-Simonds D, Lai L, Watt SR, Denaro M, Arn S, Augenstein ML, Betthauser J, Carter DB, Greenstein JL, Hao Y, Im GS, Liu Z, Mell GD, Murphy CN, Park KW, Rieke A, Ryan DJ, Sachs DH, Forsberg EJ, Prather RS, Hawley RJ (2004). Production of alpha-1,3-galactosyltransferase null pigs by means of nuclear transfer with fibroblasts bearing loss of heterozygosity mutations. Proc Natl Acad Sci U S A.

[B3] Takeuchi Y, Magre S, Patience C (2005). The potential hazards of xenotransplantation: an overview. Rev Sci Tech.

[B4] Fishman JA, Patience C (2004). Xenotransplantation: infectious risk revisited. Am J Transplant.

[B5] Martin U, Winkler ME, Id M, Radeke H, Arseniev L, Takeuchi Y, Simon AR, Patience C, Haverich A, Steinhoff G (2000). Productive infection of primary human endothelial cells by pig endogenous retrovirus (PERV). Xenotransplantation.

[B6] Patience C, Takeuchi Y, Weiss RA (1997). Infection of human cells by an endogenous retrovirus of pigs. Nat Med.

[B7] Wilson CA, Wong S, Muller J, Davidson CE, Rose TM, Burd P (1998). Type C retrovirus released from porcine primary peripheral blood mononuclear cells infects human cells. J Virol.

[B8] Tarlinton RE, Meers J, Young PR (2006). Retroviral invasion of the koala genome. Nature.

[B9] Takeuchi Y, Patience C, Magre S, Weiss RA, Banerjee PT, Le Tissier P, Stoye JP (1998). Host range and interference studies of three classes of pig endogenous retrovirus. J Virol.

[B10] Wilson CA, Wong S, VanBrocklin M, Federspiel MJ (2000). Extended analysis of the in vitro tropism of porcine endogenous retrovirus. J Virol.

[B11] Oldmixon BA, Wood JC, Ericsson TA, Wilson CA, White-Scharf ME, Andersson G, Greenstein JL, Schuurman HJ, Patience C (2002). Porcine endogenous retrovirus transmission characteristics of an inbred herd of miniature swine. J Virol.

[B12] Harrison I, Takeuchi Y, Bartosch B, Stoye JP (2004). Determinants of high titer in recombinant porcine endogenous retroviruses. J Virol.

[B13] Bartosch B, Stefanidis D, Myers R, Weiss R, Patience C, Takeuchi Y (2004). Evidence and consequence of porcine endogenous retrovirus recombination. J Virol.

[B14] Ericsson TA, Takeuchi Y, Templin C, Quinn G, Farhadian SF, Wood JC, Oldmixon BA, Suling KM, Ishii JK, Kitagawa Y, Miyazawa T, Salomon DR, Weiss RA, Patience C (2003). Identification of receptors for pig endogenous retrovirus. Proc Natl Acad Sci U S A.

[B15] EST Profile Viewer. http://www.ncbi.nlm.nih.gov/UniGene/.

[B16] Tailor CS, Nouri A, Zhao Y, Takeuchi Y, Kabat D (1999). A sodium-dependent neutral-amino-acid transporter mediates infections of feline and baboon endogenous retroviruses and simian type D retroviruses. J Virol.

[B17] Rasko JE, Battini JL, Gottschalk RJ, Mazo I, Miller AD (1999). The RD114/simian type D retrovirus receptor is a neutral amino acid transporter. Proc Natl Acad Sci U S A.

[B18] Olah Z, Lehel C, Anderson WB, Eiden MV, Wilson CA (1994). The cellular receptor for gibbon ape leukemia virus is a novel high affinity sodium-dependent phosphate transporter. J Biol Chem.

[B19] Mendoza R, Anderson MM, Overbaugh J (2006). A putative thiamine transport protein is a receptor for feline leukemia virus subgroup A. J Virol.

[B20] Kavanaugh MP, Miller DG, Zhang W, Law W, Kozak SL, Kabat D, Miller AD (1994). Cell-surface receptors for gibbon ape leukemia virus and amphotropic murine retrovirus are inducible sodium-dependent phosphate symporters. Proc Natl Acad Sci U S A.

[B21] Tailor CS, Lavillette D, Marin M, Kabat D (2003). Cell surface receptors for gammaretroviruses. Curr Top Microbiol Immunol.

[B22] Marin M, Tailor CS, Nouri A, Kabat D (2000). Sodium-dependent neutral amino acid transporter type 1 is an auxiliary receptor for baboon endogenous retrovirus. J Virol.

[B23] Eiden MV, Farrell K, Wilson CA (1994). Glycosylation-dependent inactivation of the ecotropic murine leukemia virus receptor. J Virol.

[B24] Miller DG, Miller AD (1993). Inhibitors of retrovirus infection are secreted by several hamster cell lines and are also present in hamster sera. J Virol.

[B25] Miller DG, Miller AD (1992). Tunicamycin treatment of CHO cells abrogates multiple blocks to retrovirus infection, one of which is due to a secreted inhibitor. J Virol.

[B26] Tailor CS, Nouri A, Kabat D (2000). Cellular and species resistance to murine amphotropic, gibbon ape, and feline subgroup C leukemia viruses is strongly influenced by receptor expression levels and by receptor masking mechanisms. J Virol.

[B27] Johann SV, van Zeijl M, Cekleniak J, O'Hara B (1993). Definition of a domain of GLVR1 which is necessary for infection by gibbon ape leukemia virus and which is highly polymorphic between species. J Virol.

[B28] Eiden MV, Farrell KB, Wilson CA (1996). Substitution of a single amino acid residue is sufficient to allow the human amphotropic murine leukemia virus receptor to also function as a gibbon ape leukemia virus receptor. J Virol.

[B29] Marin M, Tailor CS, Nouri A, Kozak SL, Kabat D (1999). Polymorphisms of the cell surface receptor control mouse susceptibilities to xenotropic and polytropic leukemia viruses. J Virol.

[B30] Yoshimoto T, Yoshimoto E, Meruelo D (1993). Identification of amino acid residues critical for infection with ecotropic murine leukemia retrovirus. J Virol.

[B31] Tailor CS, Takeuchi Y, O'Hara B, Johann SV, Weiss RA, Collins MK (1993). Mutation of amino acids within the gibbon ape leukemia virus (GALV) receptor differentially affects feline leukemia virus subgroup B, simian sarcoma-associated virus, and GALV infections. J Virol.

[B32] TMHMM server v.2.0. http://www.cbs.dtu.dk/services/TMHMM.

[B33] NetNGlyc 1.0 software. http://www.cbs.dtu.dk/services/NetNGlyc.

[B34] Pizzato M, Marlow SA, Blair ED, Takeuchi Y (1999). Initial binding of murine leukemia virus particles to cells does not require specific Env-receptor interaction. J Virol.

[B35] Chung M, Kizhatil K, Albritton LM, Gaulton GN (1999). Induction of syncytia by neuropathogenic murine leukemia viruses depends on receptor density, host cell determinants, and the intrinsic fusion potential of envelope protein. J Virol.

[B36] Wang H, Kavanaugh MP, North RA, Kabat D (1991). Cell-surface receptor for ecotropic murine retroviruses is a basic amino-acid transporter. Nature.

[B37] Gemeniano M, Mpanju O, Salomon DR, Eiden MV, Wilson CA (2006). The infectivity and host range of the ecotropic porcine endogenous retrovirus, PERV-C, is modulated by residues in the C-terminal region of its surface envelope protein. Virology.

[B38] Watanabe R, Miyazawa T, Matsuura Y (2005). Cell-binding properties of the envelope proteins of porcine endogenous retroviruses. Microbes Infect.

[B39] Chiang CY, Pan YR, Chou LF, Fang CY, Wang SR, Yang CY, Chang HY (2007). Functional epitopes on porcine endogenous retrovirus envelope protein interacting with neutralizing antibody combining sites. Virology.

[B40] Currie GA, Gage JO (1973). Influence of tumour growth on the evolution of cytotoxic lymphoid cells in rats bearing a spontaneously metastasizing syngeneic fibrosarcoma. Br J Cancer.

[B41] Neil S, Martin F, Ikeda Y, Collins M (2001). Postentry restriction to human immunodeficiency virus-based vector transduction in human monocytes. J Virol.

[B42] Ylinen LM, Keckesova Z, Wilson SJ, Ranasinghe S, Towers GJ (2005). Differential restriction of human immunodeficiency virus type 2 and simian immunodeficiency virus SIVmac by TRIM5alpha alleles. J Virol.

[B43] Towers G, Bock M, Martin S, Takeuchi Y, Stoye JP, Danos O (2000). A conserved mechanism of retrovirus restriction in mammals. Proc Natl Acad Sci U S A.

[B44] Naldini L, Blomer U, Gage FH, Trono D, Verma IM (1996). Efficient transfer, integration, and sustained long-term expression of the transgene in adult rat brains injected with a lentiviral vector. Proc Natl Acad Sci U S A.

[B45] Niwa H, Yamamura K, Miyazaki J (1991). Efficient selection for high-expression transfectants with a novel eukaryotic vector. Gene.

[B46] Jeffs SA, McKeating J, Lewis S, Craft H, Biram D, Stephens PE, Brady RL (1996). Antigenicity of truncated forms of the human immunodeficiency virus type 1 envelope glycoprotein. J Gen Virol.

[B47] Entrez protein database. http://www.ncbi.nlm.nih.gov/entrez.

[B48] Thompson JD, Higgins DG, Gibson TJ (1994). CLUSTAL W: improving the sensitivity of progressive multiple sequence alignment through sequence weighting, position-specific gap penalties and weight matrix choice. Nucleic Acids Res.

