# Supplementary material for: Differential resistance to cell entry by porcine endogenous retrovirus subgroup A in rodent species
Source: Retrovirology. 2007 Dec 14;4:93. doi: 10.1186/1742-4690-4-93 (PMC2241639; doi:10.1186/1742-4690-4-93)
Supplement: Additional File 1 — PERV-A receptors cell surface expression. Expression of C-terminal HA-tagged PAR constructs in QT6 cells was demonstrated by flow cytometry analysis following surface immunostaining with an anti-HA antibody. [file 1742-4690-4-93-S1.pdf]

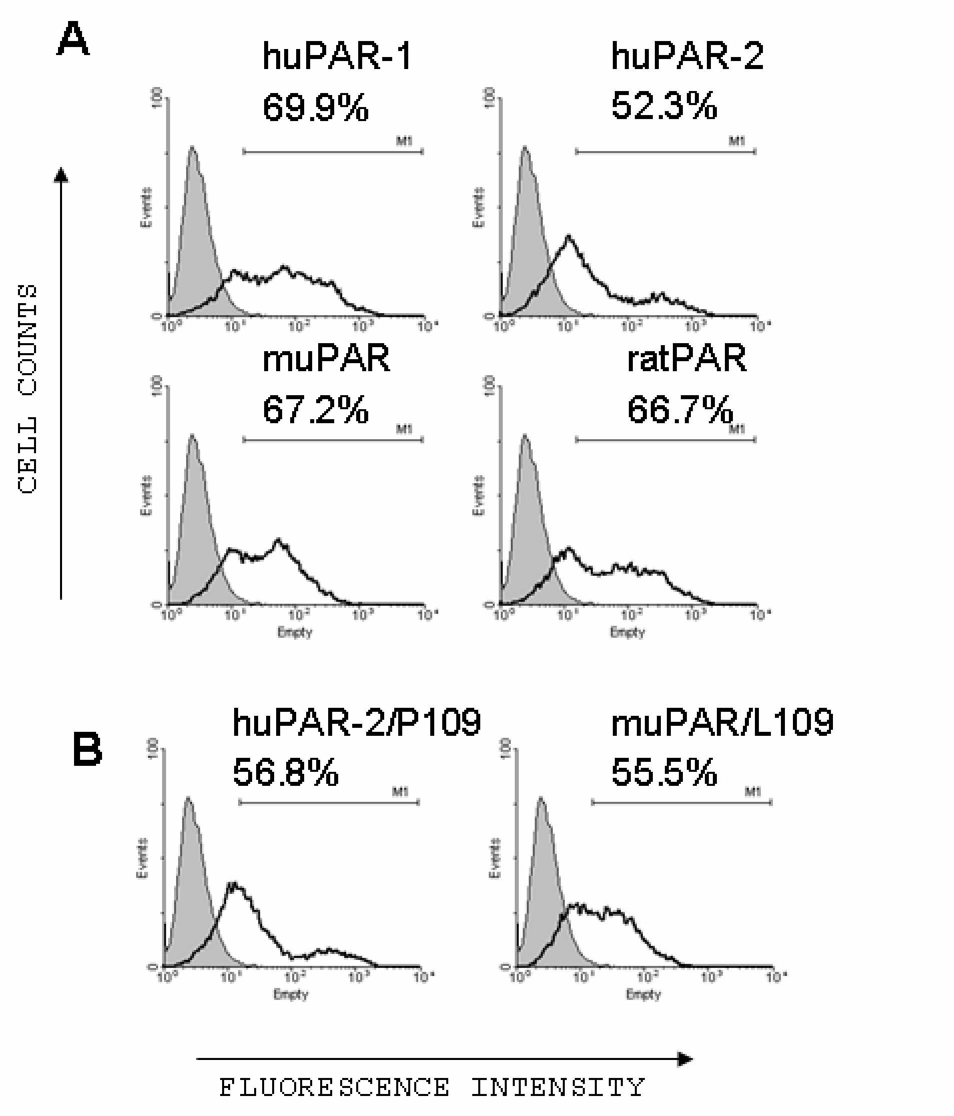

Fig S1. PERV-A receptors cell surface expression. C-terminal HA-tagged wild-type PARs (**A**) or chimeric constructs (**B**) were delivered into quail QT6 cells using an MLV-based retroviral vector. Expression of the constructs were assessed 48 hours later by immunostaining with an anti-HA antibody and a PE conjugated anti-mouse IgG secondary antibody. The cells were then processed by flow cytometry. Expression of PARs and derived constructs was also verified one and two weeks post-transduction with no difference in the profiles shown in these histograms.
