# Supplementary material for: Differential resistance to cell entry by porcine endogenous retrovirus subgroup A in rodent species
Source: Retrovirology. 2007 Dec 14;4:93. doi: 10.1186/1742-4690-4-93 (PMC2241639; doi:10.1186/1742-4690-4-93)
Supplement: Additional File 2 — RatPAR function as PERV-A receptor depends on its expression on cell surface. PERV-A infection was measured in quail QT6 cell clones expressing different levels of ratPAR. [file 1742-4690-4-93-S2.pdf]

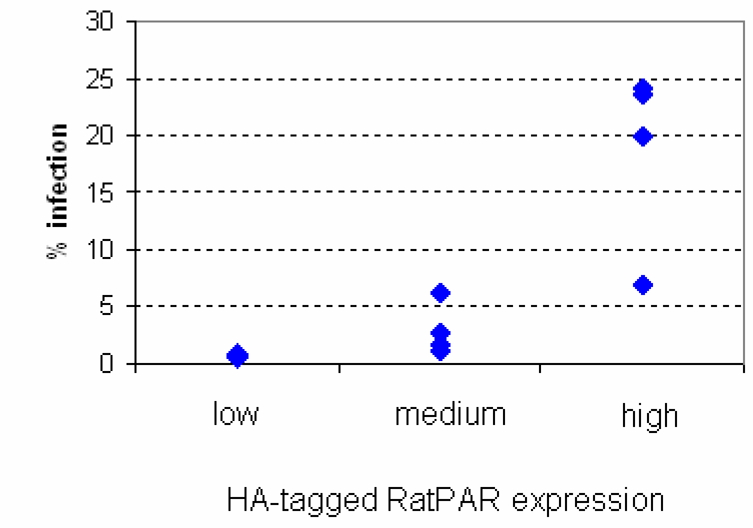

Fig S2. RatPAR function as PERV-A receptor depends on its expression on cell surface. Quail QT6 cells were transduced with retroviral vector carrying HA-tagged ratPAR gene and single clones were isolated. For each individual QT6 clones, the amount of ratPAR on cell surface was determined by immunostaining using anti-HA antibody. The clones were divided according to mean fluorescence intensity (MFI), i.e. RatPAR expression: low (MFI:10-60), medium (60-200) and high (>200). The results were correlated with the efficiency of EGFP(PERV-A) infection. The graph is representative of two independent experiments
